# The 5-Minute Campus

**DOI:** 10.3390/ijerph20021274

**Published:** 2023-01-10

**Authors:** Sascha Naomi Jansz, Mark Mobach, Terry van Dijk

**Affiliations:** 1 Department of Spatial Planning and Environment, Faculty of Spatial Sciences, Rijksuniversiteit Groningen, 9712 CP Groningen, The Netherlands; 2 Research Group Facility Management, Research Centre for Built Environment—NoorderRuimte, Hanze University of Applied Sciences, 9747 AS Groningen, The Netherlands; 3 Research Group Spatial Environment and the User, The Hague University of Applied Sciences, 2521 EN The Hague, The Netherlands

**Keywords:** 5-minute campus, interactions, campus design, unplanned meetings, proximity, facilities

## Abstract

As campuses wish to stimulate interactions among different campus users, we aim to identify why some locations are successful in fostering unplanned meetings while others are not. This can help campus managers, directors, and other practitioners to optimize their campus to facilitate unplanned meetings between academic staff and companies. Findings of a previous survey were discussed in five focus groups, which were transcribed and thematically coded. Three separate theme groups were identified: function (food, drinks, events, work, facilities), space (distance, experience, accessibility, characteristics), and organization (coherence, culture, organization). Time was an overarching constraint, influencing all other themes. There were three natural moments for unplanned meetings: during short breaks, lunch breaks, and events. The outcomes suggest a 5-minute campus as the environment of interaction; a campus where natural moments, locations, and travel time for unplanned meetings are designed and aligned: (1) under 5 min walking for short workplace breaks, (2) approximately 5 min travel time for lunch breaks, and (3) over 5 min travel time for events, depending on the event length and anticipated knowledge gain.

## 1. Introduction

Education, research, and valorization: these are the three main objectives of Dutch universities [1]. To stimulate knowledge valorization, a triple helix of academic-industry-government relationships is often used, as described by Etzkowitz [2]. In part, universities do so by attracting companies to their campuses [3,4,5]. One thing that sets any campus apart from a more general collection of buildings is that campuses seek to create meeting places for all campus users, regardless of their organizational background. Such meeting places are intended to foster unplanned meetings between these different user groups, which in turn may lead to collaboration and knowledge sharing [6]. These unplanned meetings are therefore seen as an important pre-requisite for innovation and considered essential to achieving university valorization goals [7,8].

Many campus directors would like to optimize their campuses by facilitating these unplanned meetings, but providing the required attractive and versatile range of accommodations and facilities is a puzzle for many [3,9]. In city design, concepts of a 5-, 10-, 15-, or 20-minute city-limiting the travel distance to amenities to the mentioned time limits—have been suggested to improve social cohesion as well and health and sustainability [10,11,12,13]. These concepts are also being implemented by municipalities, using walkability analysis to assess their current city design [14]. The campus may also benefit from such an approach, although it is not currently applied in practice on campuses in the Netherlands. Additionally, as the campus is primarily a work environment (although some do include living on campus as well) it is unknown what time limit, and to which amenities, would be appropriate.

Our study aims to inform directors and designers about effective campus designs, i.e., ones that foster unplanned meetings.

The following definition of an unplanned meeting is used: “a serendipitous meeting between at least two people as a direct result of coincidental mutual visual contact”. “Intentional unscheduled visits” (the initiator of the conversation walked up to someone) are not included as described by Brown [15] (cited by [16] Page 878). The focus is on conversations “initiated after coincidental visual contact” (partners happened to see each other and started talking). As participants at (networking) events do not know which other participants will be attending and/or if there will be an opportunity to speak to them, these unplanned meetings are included. This opportunity has the capacity to create weak ties [17] between people, which can potentially be strengthened and may lead to collaboration over time. Digital meetings are not included in this study.

Van Andel describes serendipity as “the art of the unsought finding” [18]. Outside of normal workplaces, facilities such as coffee machines, hallways, and seats can encourage frequent unplanned interactions, and by doing so, can help bringing people together [16,19]. As described by Björneborn, serendipity cannot be engineered or designed per se, but the affordances for serendipity can [20]. However, this only applies to the designer’s point of view; seen from the from the user’s perspective, in order to be serendipitous, serendipity is always unplanned [21] (page 23). 

Following Gibson’s theory of affordances, we do not perceive an environment’s or object’s qualities, but the affordances it offers us, i.e., a handle affords grasping [20,22]. Furthermore, affordances may relate to privacy (enable people to control the boundaries of a conversation), propinquity (allow people to come into unplanned contact with others) and social designation (make people aware that it is socially acceptable to stop and talk to each other), according to Fayard and Weeks [21]. In their study architectural elements, geographical elements, and functional elements had a positive or negative influence on privacy and propinquity [21]. 

With specific but yet unclarified mechanisms, the built environment is expected to influence unplanned on-campus meetings. However, the extent to which different settings afford informal interactions may vary widely. These types of interaction will be fostered in some settings, but not in others [21].

All this is framed by different motivators and constraints, dependent on the context of the campus. Motivators are ‘the reason for a behavior’ [23] (page 249). Motivation can be intrinsic, referring to the satisfaction of performing an action for its own sake, or extrinsic, based on any sort of external reward or punishment [24]. Motivators make us participate in certain behavior, while constraints, also known as barriers, “stifle” certain behavior [23,24]. In studies on environmental behavior, this interaction between motivation and constraints has been discussed considerably. 

**Figure 1:**
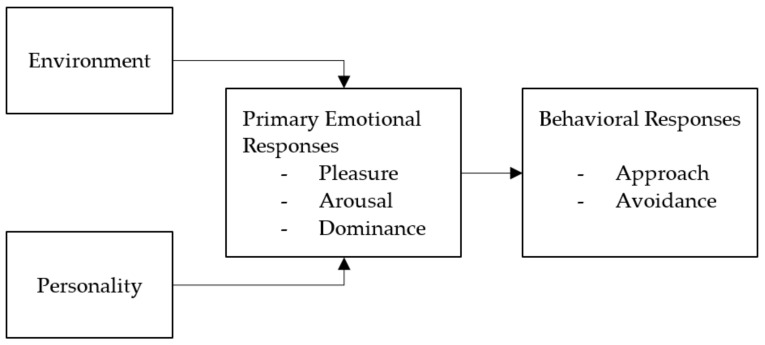
Classic environment model ([29], page 8). Reprinted from An Approach to Environmental Psychology by Albert Mehrabian and James A. Russell, reprinted courtesy of The MIT Press.

These constraints can be structural, social, or psychological. This means that although removing structural constraints is important, it is not sufficient [25]. However, constraints do not always prevent an action. When the motivators are strong enough, constraints may be negotiated by campus users [26] (cited by [24] Page 363) and acted upon accordingly. Additionally, literature suggests that highly motivated people perceive fewer constraints [27,28] and in leisure research, motivation has been considered to be an important factor to overcome leisure constraints [24]. Some motivators and constraints can be highly personal—some people enjoy going out to meet new people, while others do not—whereas others are more general [24]. In other words, approach and avoidance behaviors are influenced by both the environment and personality traits, as described by Mehrabian and Russell (see Figure 1, [29] page 8).

Unplanned meetings perpetuate networking, which can be defined as “a form of goal-directed behavior, both inside and outside an organization, focused on creating, cultivating, and utilizing interpersonal relationships” [30]. Gibson, Hardy, and Buckley describe three levels of precursors (or antecedents) to networking that influence networking: individual, job, and organizational levels. On the individual level they name personality, self-esteem, attitudes toward workplace politics, marital status, education, and gender. On the job level these are hierarchical position, type of job, and hours per week. (An individual’s higher hierarchical position in the organization is associated with larger networks and more active networking behaviors.) On the organizational level these are the organizational culture and the industry [30]. They describe the influence of the organizational culture as follows: “Many decisions and policies implemented by organizations will have important implications for the extent and frequency with which their employees’ network. For example, an organization with a collaborative and open culture will be more likely to facilitate networking behaviors than one in which employees are competitive and mistrustful of one another.” [30] page 155.

This paper uses as its point of departure the survey data of a previous paper by the authors [31]. In this related survey study, academic staff of three Dutch university campuses was asked to indicate locations on a map of their campus, where they originally met people from companies who later became partners. As mentioned above, these first, unplanned meetings are part of the basis for collaboration and innovation [6]. Additionally, they were asked about the spatial characteristics of these places and the services that had contributed to their interactions. The locations and their characteristics were then analyzed through a spatial analysis, principal component analysis and linear regression resulting in four principal components for services (Relax, Network, Proximity, and Accessibility) and three principal components for locations (Aesthetics, Cleaned, and Indoor Environment). A comparison between locations in a respondent’s own building (residents) and in other buildings (visitors) was made. After all, when people are on the move, possibly also venturing into other buildings, more opportunities for unplanned meetings with new persons may arise. Looking at the indicated locations on the map, it was also clear that the spatial characteristics on their own did not explain why some buildings were a popular destination and others were not. To further understand this difference and to learn why people move around on campus, this study focuses on the differences between users and related explanations in a focus group discussion.

## 2. Materials and Methods

The authors conducted a related survey of 443 academic staff members on three Dutch university campuses [31]. To ensure that interrelationships could be discussed and in-depth reasons could surface [32], a qualitative approach in the form of a focus group was chosen to better understand why respondents would choose one location over another. Additionally, as the survey study lacked sufficient response from company employees, extra efforts were made to include these employees. This was successful. Moreover, the results of the survey were presented and discussed in the focus groups as a baseline and trigger for discussion. 

### 2.1. Participants

As described by Brown [33], focus groups are helpful in identifying thoughts, perceptions, and impressions, especially when the interest group may be difficult to access. As interviews would not have facilitated discussion among the participants, which allows differences between personal tastes as well as organizational background to come forward, and the company representative had indicated that a workshop would have been too time consuming, a focus group set up was most appropriate. Therefore, five focus groups were questioned to generate a more in-depth, qualitative understanding of the survey results. This approach, to add richer meaning to the close-ended survey questions and to ask questions related to experiences and behavior, opinions, feelings, and general background, was described by Rowley [34] and Rosenthal [35], respectively. 

Participants were contacted based on their indication in the survey that they were willing to participate in further research. Additionally, to gain participation from companies as well, participants of the ‘Campus coffee talk’ (online) event, organized by Campus Groningen, were also invited. The focus group sessions took place in June 2021. As the statistical analyses of the survey results showed that there were no significant differences between the three different participating campuses, and respondents from one campus gave similar responses to those from the other campuses [31], only participants from the Zernike campus in Groningen were included in the focus groups. Moreover, this campus had the most respondents that indicated willingness to participate in the follow-up research, as well as being the lead author’s own campus, creating the opportunity to include campus management and the local business association in recruiting participants from companies. 

**Figure 2:**
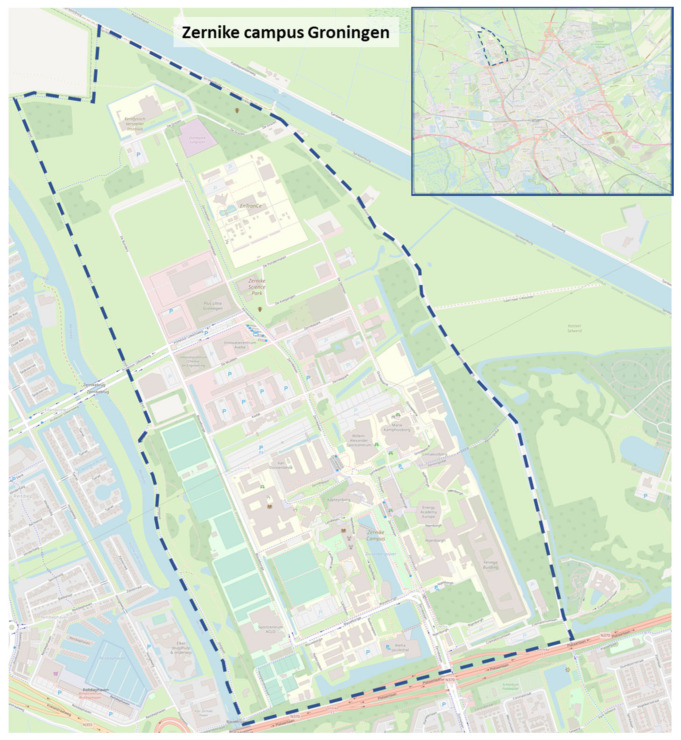
Zernike campus [37]. Map data copyrighted OpenStreetMap contributors and available from https://www.openstreetmap.org, accessed on 13 December 2022.

**Figure 3:**
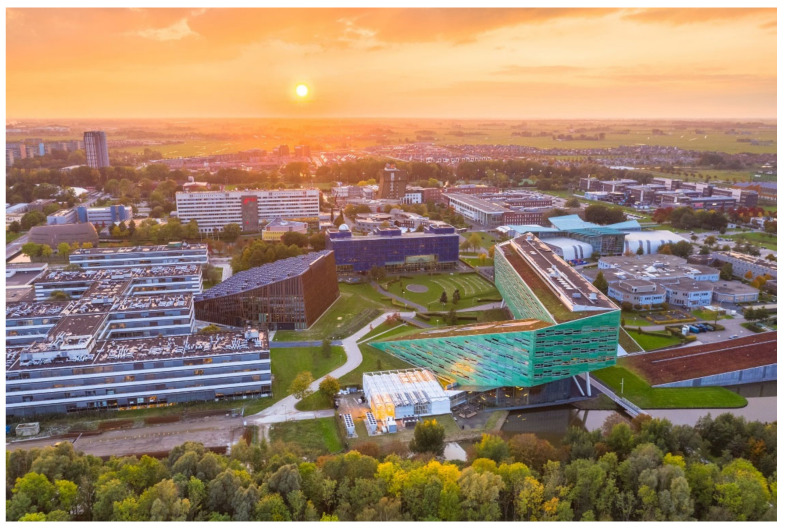
Zernike campus [38].

The Zernike campus in Groningen is a university campus. This means that the University of Groningen and the Hanze University of Applied Sciences are the main campus users. The campus is located north of the city of Groningen, at the edge of the city, and is 126 hectares. The University of Groningen first developed in the inner city of Groningen and later moved part of its operations to the Zernike campus. The University of Groningen has 34,000 students and aims to connect education and research with sustainable and economic processes through three strategic themes: Energy, Healthy Aging, and Sustainable Society [36]. See Figure 2 for a campus map and Figure 3 for a photo impression 

To ensure sufficient representation from companies, a 50% company, 50% knowledge institution representation rate was chosen for the focus groups (see Table 1). To allow for interaction between these two participant groups, three out of the five groups were mixed. Due to COVID-19 restrictions, the focus group discussions took place online. To ensure that all participants would be able to actively participate and to prevent them from feeling overwhelmed by too many faces on the screen, groups were set at a maximum of six participants per group. The focus group facilitator also ensured all participants were included in the discussion before moving on to the next question. 

There were participants from nine different companies (five directors/CEOs, five managers, and one coordinator; five female, six male). There were eight participants from the University of Groningen (two full professors, three assistant professors, one researcher and one scientific coordinator; two female, six male) and three participants from the Hanze University of Applied Sciences (one Ph.D. and two researchers; one female, two male). 

### 2.2. Focus Group Protocol

All focus groups began with a short introduction by the lead author of this paper, explaining all important definitions (see introduction), highlighting the main survey results, and explaining our aim to find the in-depth reasons of the behavior found in the survey. The interview protocol included four propositions, specifically worded to generate discussion, which were based on the survey results and introduced by the researcher. The propositions were: (1) ‘Even if the quality of the building where I work is low, I still prefer to stay here for interactions.’; (2) ‘The only reason to move to a different building is if there are people there with specific knowledge I need (e.g., companies or special expertise).’; (3) ‘When I go somewhere for coffee or lunch, it is one of the main moments in which I have unexpected meetings.’; and (4) ‘The main reason I meet people outside my building is for an event or meeting.’

The focus groups were semi-structured to ensure each proposition could be addressed by each participant, but also to leave room for participants to add context [39]. All preparations (invitation, briefing, etc.) were standardized and the same protocol was used to ensure compatibility. All focus groups were conducted online through Google Meet, due to COVID-19 restrictions being in place at the time. Participants were asked to answer based on their on-campus experiences before these restrictions were in place. When experiences during the COVID-19 pandemic came up, the discussion was redirected to experiences from before the pandemic, but this happened only occasionally. Everyone introduced themselves at the start of the meeting. The focus groups were planned to last 1 h, with an additional 30 min of overflow time in which additional questions from participants, also outside the scope of the focus group discussion, could be answered.

### 2.3. Analysis

Informed consent was obtained from all participants and all communication of the focus groups was recorded and transcribed. In all groups, all participants confirmed to be comfortable in understanding both English and Dutch, but some participants preferred to express themselves only in one language. As the lead author is fluent in both languages the transcription was made verbatim in the language used by the participant at that time. 

Using ATLAS.ti V22 software [40], an inductive thematic analysis was conducted [41]. The thematic analysis consisted of five steps, as described by Braun and Clarke [41]: data familiarization, initial coding, searching themes, reviewing themes, and defining and naming themes. The themes were grouped by the lead author based on the focus group data. As themes emerged, the lead author assigned working names to the themes, which were later finalized when themes were grouped into overarching categories. During this process the lead author discussed the analysis with the other members of the team, and all agreed on the final results. 

The analysis was also used to derive quotations, which are used to illustrate the emerging themes. 

Finally, a concept derived from the data were fed back to the participants with a confirmatory question: Does this indeed match your experience? 

## 3. Results

**Figure 4:**
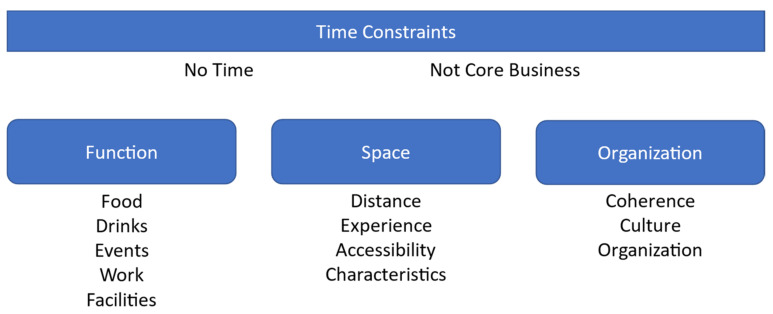
Thematic map.

In this section, the findings are presented according to four themes: (1) time constraints, (2) function, (3) space, and (4) organization (see Figure 4). Quotations are used to illustrate the themes, which are described in more detail below. When words are not part of the original quotation these are stated in square brackets [interpretation authors]. When participants were speaking Dutch, these quotations have been translated to English by the authors.

### 3.1. Time Constraints

The analysis revealed that there was one overarching theme, which affected all the others: Time constraints. This consists of two parts: (1) having no time in general, and (2) having no time for unplanned meetings specifically as this is not considered ‘core business’. Participants clearly stated that although all the other aspects (see below) were also important, eventually many answers were repeatedly related to the experienced time constraints in their daily work. For example, when asked why some buildings in the survey had attracted respondents from other buildings and to give plausible explanations and motivations for traveling between these buildings on campus, they said the following:

*“I feel that people are often running short on time. You don’t have much time to move around buildings which easily takes you 15 min. And many people don’t want to take the time, don’t have the time to do that. So the meetings happen around your actual workplace, the coffee machine, the copy machine, or on the way to your lunch break. So, it’s the proximity.” (R01)*

*“These movement activities [moving between buildings], they depend on purpose, on time of the day, and on proximity of the building to the office, because everything is related to limited time.” (R02)*

In short, they said that time is already limited, it can be hard to find the time to go on a break, let alone venture too far from the workplace. In addition, even though unplanned meetings are important to build your network and find new work partners they are not considered urgent:

*“It’s not core business.” (R03)*

*“I don’t mind walking, but that’s not work.” (R04)*

When asked how much time they would have available/be willing to spend to move around campus or attend an event, participants indicated there were three different types of spatial-temporal activities: around the workplace, the lunchtime (or coffee break), or the attendance of an event.

For each, different travel times seem appropriate, and these times also include the travel time within their own building. Around the workplace this should be less than 5 min, as days are often fully planned with meetings. For a lunch break, participants reported to have a total of 30 min available. As this includes ordering lunch, waiting for your order, and eating your lunch, this creates a very tight schedule if traveling between buildings is involved. Finally, for events the content and length of the event is important:

*“I think it depends on how long the meeting or event will be. So, if you have an event or meeting for one hour, then you are not willing to walk 15 min. But if it’s … a 3- or 4-h session I don’t mind. So that is also, it should be a little bit in relation to the meeting.” (R05)*

Time also includes the travel time within their own building, which can be limiting.

*“I realized that when I started working on the 8th floor: ‘Oh, this takes basically quite some time, because you have to wait for the elevator and of course on the way back too.” (R03)*

### 3.2. Function

A main theme revealed in the analysis was that due to the time constraints, participants felt that they needed an attractor to venture out of their daily routine. It needs to have a clear, specific, and appropriate function:

*You kind of need to pull people towards a place. That has a specific thing going on. Otherwise, they’re just out there walking the same square that they do for lunch every day, and then they go home. (R07)*

*And if you don’t have some attractor like that, then I would even say [you stay] only on your own floor or your own wing. Outside of that you never meet anyone. (R08)*

The following themes related to function were revealed in the analysis of the focus group discussions: food, drinks, events, work, and facilities. Different participants mentioned different preferences, but catering, and more specifically lunch and coffee, were very prominent.

*The main reason [I meet people outside my own building] is food and coffee. It remains the main reason. Primary life necessity. (R07)*

A central facility for lunch (and/or conferences) was mentioned frequently as a strong attractor with a higher chance of meeting people, as long as it was not too far away. However, a few downsides were also indicated: this could also generate a lot of traffic around lunchtime and people tend to all walk in the same direction, so you would not meet as many people after all. 

Participants also indicated they meet different types of people at different locations.

*I would say the coffee and lunch is more for the smaller circle, for instance, unplanned meetings with colleagues or [other] people you know. And [when you meet at an event] it is an unplanned meeting with a larger circle, so people you don’t know yet or people you don’t regularly meet. (R08)*

The quality of the food or drinks, and especially good quality coffee, was also a strong attractor. However, due to the long distances from the companies to the food court, company employees felt a barrier to enjoy these facilities, even though they do not have something similar close by.

*[In the north of the campus, where most companies are located] there is not much to experience in the form of a canteen or places where you can sit. Or can mingle with other people. Because that is a bit further back on campus. So, to walk to the food court, you could do that. But it is a long way. You could. But you don’t. (R03)*

For drinks, not only (good quality) coffee was important, the possibility to have a drink after work also came up repeatedly. There is a trend that people quickly leave the campus after work and having a bar or restaurant type function available might help mitigate that. Bars are mentioned as a place where many collaborations start; this is also linked to organizational culture ‘How things are done around here’ [42] (see also below).

*You go there because there are facilities that all people and companies on the campus can use. Like a conference center, or lunch facilities, or the coffee bar, or a real bar … things like that. I mean something useful or nice that you use during or at the end of your working day. And then it starts to live then, that’s where you meet people. (R08)*

Several types of events were mentioned by the participants: content (work related) events, networking events, cultural events, and fun/social events. Participants clearly indicated that events are part of what defines a campus.

However, once again, time was the main constraint mentioned by participants when deciding to attend an event or not, followed by the content or subject matter of the event. (See also coherence in Section 3.4.)

*I regularly skip meetings or events because I think I cannot learn something from it, or I cannot bring in my knowledge in a proper way. (R06)*

Content events, such as conferences or symposia, are mentioned as a real attractor, because the events’ subject matter ensures the presence of people with similar interests.

*When there’s an event you are already with people that have some kind of same interest, then it is easier to start a conversation. Sometimes when I go for lunch or for coffee, you’re a little bit … You go, you take it, and you leave, you know. (R12)*

Moreover, content related events, networking events are mentioned. As these are organized specifically to get to know new people, and are often organized around a theme or topic, they create an atmosphere where people feel free to make new connections.

*If you go to an event, you are sure you will meet other people. And you’re also a bit open to it because you are ready to meet other people. That’s usually the reason to go to an event also. (R13)*

This is a very important function for participants, as they are often looking to find those people that can help then on a particular topic. 

Additionally, fun or social events are also mentioned as great opportunities for interactions. Participants indicate that after-work drinks, hackathons, (Christmas) markets, and company outings are important events where they meet new people.

*It’s events from [the staff association] that organizes events, and these kinds of activities that you attend. And there, spontaneous interactions happen. (R01)*

Finally, cultural activities, such as music, art, going to a movie, or engaging in other activities that generally take place after working hours are mentioned. However, as these are the least work related, these are very much dependent on the organizational culture (see below). 

Participants also indicated that it is important to have the required facilities to be able to organize these events. For example, about social events:

*There’s no space to kind of carry these types of activities. There’s is no restaurant or organization that has a stage. On the campus to do any of this. … There is no place to book a room, to have a drink with your department or company to celebrate something. So, what this campus misses entirely is celebrations. There is nothing there to support that. (R07)*

This is also what connects the factors mentioned above, to be able to be an attractor, the proper facilities have to be there. These include facilities for food, drinks, events, or sports, but also (shared) research facilities.

*As I said before, I used to go for lunch at the food court because it has the facilities. If there are facilities around which attract me, then I’ll go there. (R10)*

Other functions that might be used as attractors mentioned by the participants were: cultural activities, a stage, a proper restaurant, a weekly market, food trucks, vendors, after work drinks, and celebration spaces. 

Aside from all the attractors mentioned above, work remained the most frequent reason participants had met new work partners. Planned meetings or teaching were mentioned as the most frequent, as they are a daily part of the job. At these planned meetings, participants would then be introduced to other participants, often by their co-workers. 

### 3.3. Space

Once a participant is in a certain location, the spatial characteristics of that location are important as well. The following themes are mentioned by participants: distance, experience, accessibility, and spatial characteristics. 

Again, time, the overarching constraint, has implications: the distance is mentioned as the main spatial constraint by participants. 

The larger the distances, the lower the chances that people will run into each other:

*We would love to have the situation where a Ph.D. student, by accident, bumps into someone from a company. And they talk about and ‘hey, are you doing this, are you doing that?’ But that is virtually absent as far as I know. Because the distances are big and of course you can’t chance that really. The physical distances. But also, because these facilities are a bit spread out and a bit small and not so handy often. (R03)*

Creating the right facilities as attractors can mitigate this problem, as well as creating a nice and safe route to walk or bike. 

Participants also mentioned that the experience is important. A space has to have a certain feel, to make sure it is comfortable and a location where you would be open to meet with others. Aside from having the required facilities, a location’s experience can also make it an attractor.

*Of course, I tried to find the place that is kind of attractive then. So, you have a nice coffee corner or perhaps we can sit outside. (R06)*

Similarly, when a location is unattractive, people will be unlikely to go there or when they do, will not stay for very long. However, finding a location attractive or not can be dependent on personal taste. Participants mentioned that the experience is important to create a surrounding that you can be proud of. 

Another issue that was brought up by several participants was the campus’s accessibility and wayfinding. Not all locations are publicly accessible, or they seem inaccessible as they look too uninviting or hostile, and it can be hard to find your way on campus. This is often because companies want to protect their confidentiality, but it also means that there are no opportunities for unplanned meetings with other campus users. In addition, even when facilities are publicly accessible, this is not always clear to the campus users.

*It’s hidden! Don’t hide it, pull it out into the open! Cause that’s a problem, right? If you don’t put it out into the open, then it’s not there. Basically. (R07)*

Finally, participants mentioned several spatial characteristics that were important contributors to their on-campus interactions. These factors are scaled: when implemented well they facilitate interactions, when they are not implemented (well) they are barriers to interaction. The following were mentioned by participants: 

Infrastructure: Large infrastructures, such as wide roads, can create barriers that campus users find harder to cross. On a smaller scale, once inside a building, having to cross multiple doors or having to travel to higher floor levels, is also seen as a barrier to interaction. Human scale infrastructure such as walking and biking paths encourage users to travel across the campus. 

Scale and diversity: when places are crowded participants tend to leave sooner and feel less comfortable. However, if spaces are well set up with plenty of places to sit and enjoy being in the space, the chances of interaction with other campus users increases. Some participants prefer larger open spaces, while others do not. Providing different types of spaces or sectioning a larger open space into different parts could mitigate this problem. 

Privacy: it can be hard to find the right balance in privacy. On the one hand, a space should be inviting so that people feel free to enter and remain in the space. On the other hand, exchanging knowledge and getting to know new people requires some privacy as well, as participants reported that confidentiality and some degree of spatial enclosure by creating a separate space are important.

Noise: similar to privacy, the level of noise is tricky to balance. Some participants indicate a certain level of background noise is enjoyable, while others feel it quickly becomes too noisy to really engage in conversations, especially in larger open spaces.

Temperature: creating a comfortable indoor environment is important. Participants mention going out of their way on warmer days to find a location that is more comfortable.

Vegetation: participants mention including vegetation in the design, both indoors and outdoors, reducing the amount of concrete and/or brick and including plants and larger outdoor vegetation to create more park-like spaces. 

Outdoor spaces: including outdoor spaces, especially around a body of water, possibly with a deck or terrace, is mentioned repeatedly. This gives campus users an attractive space to visit that is different from their indoor work environment.

*If I understand correctly, more seating will be installed around the pond. Then you would get such a traffic area of meeting places there. (R09)*

### 3.4. Organization

While the facilities and buildings or spaces certainly are important, the role of people and a campus organization should not be underestimated.

*[This is] all very focused on the buildings, while I think the communities and the people should be much more central. In addition, then the buildings will support that. (R11)*

However, this is not easy to organize. Campus managers do struggle with finding what they can or cannot do and what the best interventions are. An extra challenge is that there are different organizations at play on the campus. Sometimes this leads to a doubling of campus facilities, while different campus users can be unaware of the facilities at other organizations.

The following themes are mentioned by participants: coherence, culture, and organization. 

The connection on content and ensuring people actually have interesting information to share with each other was mentioned by participants in relation to events (see above), but also as part of the campus management. Creating this coherence on campus can be done in several ways. Participants mention companies on campus with a related field of interest and taking a leading role in organizing on-campus events. Incoherence could be mitigated by campus managers, but participants also indicated that companies will likely regulate themselves.

*If you have a company that just produces e.g., cookies, it’s not very interesting for them to have, or to develop, their knowledge base as strong as companies that are focused on innovation. And these companies that are actually interested in innovations, they are here for a reason, on the campus. Because you have this connection close to research [with] a similar focus. Whereas companies that don’t really search for this innovation, they probably settle somewhere else. (R08)*

Another theme that was mentioned in relation to (practically) all the other themes is the campus’ culture. A campus’ culture can have different aspects that influences the opportunities for unplanned meetings. Participants mentioned the habit of bringing your own lunch to work, not leaving your desk during break, going home straight after work, joining others at a table (or not), and inviting colleagues. Participants also attributed these to Dutch culture and indicated this may be different for expats and internationals.

*A lot of my collaborations started at bars, after work, in conferences. But here usually… it’s also part of the Dutch culture just to go home after work. And then maybe you’ll meet friends afterwards. (R10)*

Additionally, the setting is also important, changing what aspects of the campus culture are at play. As mentioned before, attending an event will make participants more open to conversations and new connections. 

Finally, a campus organization or management is mentioned as important. Often, realizing projects on campus is a long process, and implementing changes includes many different stakeholders. Campus management is therefore important to make sure it all stays on track. It can play a role in creating a lively campus, e.g., by organizing events, and encouraging campus users to use the campus’ opportunities to the fullest. However, participants also indicated that the organization could sometimes be more flexible, e.g., allowing more different vendors for catering.

However, cost is also important, and participants realized that each decision has its implications.

*Several universities are working on it and yet, are struggling with it. To arrange that properly, efficiently. On the one hand, you don’t want to have too little space, but on the other hand, you don’t want it to sit empty either. Because that just costs money. So, it’s understandable that it’s always a bit of a squeeze on all sides. (R03)*

An event organizer can be a great addition as well as a newsletter to make sure campus users know what is going on and what activities they can join. 

### 3.5. A Comparison of Findings

In the focus groups the results from the survey and the previous study in which campus directors indicated the key success factors for unplanned meetings on campuses [31] were confirmed, and the understanding of why these findings occurred were deepened. This approach also yielded additional findings. Table 2 shows an overview with the main findings from the survey study (for full survey results please see [31]), related focus group questions (phrased specifically to induce discussion), and additional findings (within protocol, but stemming from participants’ questions and possibly beyond scope). It is, therefore, an exemplary overview of these findings.

### 3.6. The 5-Minute Campus

The results revealed that time constraints were an overarching theme for campus users: as unplanned meetings with potential new partners are perceived as not being their core business, there simply is no time for this during a normal workday. However, there are ‘natural’ moments (locations) occurring throughout the day when serendipitous meetings can happen. These coincide with core business activities, such as interactions: (1) around the workplace, (2) at a lunch or coffee break, and (3) at planned meetings or events. For each of these, participants are willing to travel farther, investing more time, if there is sufficient motivation. A participant will travel farther for a high-quality lunch (e.g., at the food court) or an event that lasts longer and/or has a topic that fits well with their daily work. 

**Figure 5:**
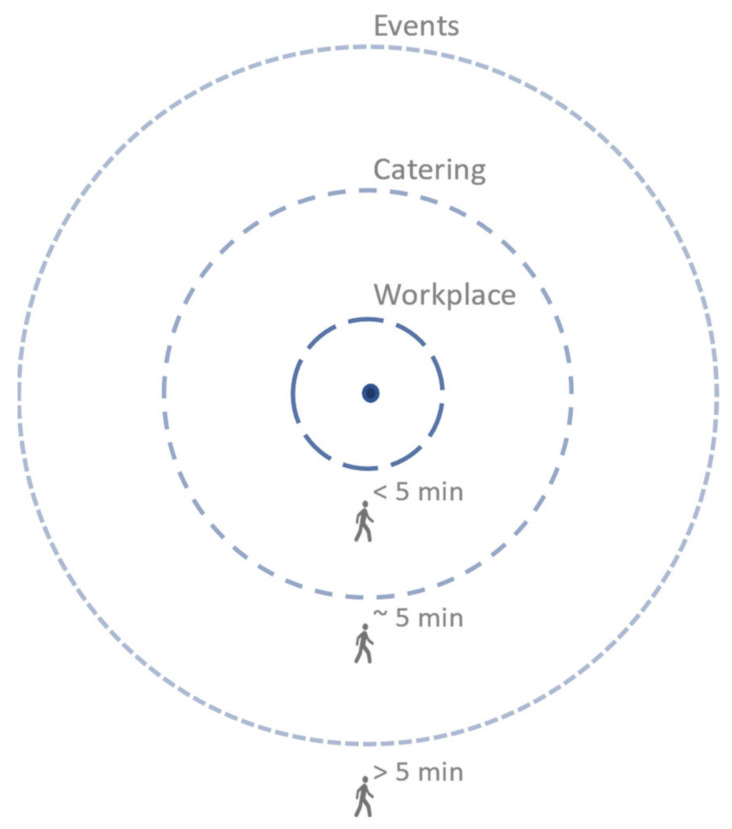
The 5-minute campus.

Participants suggest the following for different spatial-temporal interactions (see Table 3, Figure 5).

This concept was also fed back to the participants with a confirmatory question: Does this indeed match your experience? One participant could no longer be reached, because of a job change. Out of the remaining 21 participants, 19 replied (90%). Out of these 19 participants, 16 agreed (84%). Three participants disagreed (16%). Out of these three, one participant clarified that the time limit should be even lower, 5 min being the absolute maximum.

## 4. Discussion

### 4.1. Motivators and Constraints

Similar to research on environmental behavior and leisure [24,26,27,28], motivators and constraints play a large role in campus users’ behavior. On one hand, participants repeatedly mentioned that attractors are indispensable to create movement across the campus, while constraints limit the possibilities to fit certain activities in their workday. Participants clearly indicated that the more attractive an opportunity is (better coffee, interesting lecture, expectation of valuable new contacts), the more likely they are to enter a process of negotiation to tackle existing constraints. This is also true for spatial characteristics: the more comfortable or attractive a space is, the more likely that people will walk a bit further to reach it. In addition, on the other hand, the more barriers there are, such as a slow elevator with long waiting times or a large distance between the work location and the food court, the less likely it is that people will go there. In other words: campus design can be used as a supportive tool in this process of negotiating time and distance constraints. The spaces and services on a campus can create affordances to meet new people.

However, there are limits to the role campus design can play. As mentioned in the introduction, Gibson, Hardy and Buckley [30] describe three levels of antecedents for networking: individual, job, and organizational. People in a certain role, especially those higher up in the organizational hierarchy, may be more motivated to spend effort on meeting new people, helping them to successfully negotiate constraints. There may also be people who, regardless of the absence of constraints and the presence of strong attractors, still do not want to do so, e.g., as part of their personality. Moreover, the organizational culture plays a role, e.g., a collaborative and open culture will be more likely to facilitate networking [30]. Participants mentioned the presence of basic structures that encourage unplanned meetings and emphasized that these certainly should not limit people’s motivation. The fact that it was so difficult to arrange a lunch or snack on location due to the catering contracts (only contracted caterers are allowed on campus, which is expensive and very time consuming to arrange) was mentioned multiple times as a barrier to spontaneity, e.g., organizing events or smaller scale meetings.

Expectations of who participants might meet and the opportunities for sharing relevant knowledge were very important in negotiating constraints or not. This expectation of cognitive proximity (with enough in common to communicate effectively yet also with enough differences in knowledge base to learn from each other [43]) was also affected by the location type: for lunch locations participants would expect unplanned meetings with ‘weak ties’, people they know remotely [17], while for events, the expectation was to meet completely new people, enabling further expansion of their network. The lower participants’ expectations of meeting people that could add to their knowledge base, the lower their motivation was to attend an event or move a larger distance across the campus. 

### 4.2. The 5-Minute Campus

Inspired by the 5-,10-, 15-, or 20-minute city [10,11,12,13], the authors suggest that to stimulate unplanned meetings on campus, most facilities should be located within a 5-minute radius: a “5-minute campus”. This will allow users to travel around the campus at naturally occurring moments during their workday, thus increasing opportunities for meeting potential new work partners. However, a common critique on these x-minute city concepts is that acceptable travel times may vary by destination [44]. The 5-minute campus concept takes this into account by diversifying its travel time recommendations. To accommodate the three different levels of interaction, it is suggested that all workplace-related facilities (printer, coffee machine) should be situated a maximum 1–2 minutes’ walk from the user’s base location (office/desk). Higher end catering services, such as lunch options and high-quality coffee, should be a maximum 5 minutes’ walk, including the time it takes to travel within the user’s own building. For events, participation decreases when travel distance increases; 5–10 min is preferred. However, for incidental larger/longer events or events that have a high-quality content, a longer travel time is acceptable. Further research on these time limits is recommended, as the number and size of the focus groups in this study was limited. When a campus is too large to stay within the 5-minute boundary, multiple campus centers could be considered. If possible, these could be centered around different themes (e.g., health, energy, etc.) to facilitate people with a similar background using the same facilities. 

A central lunch facility was named multiple times by the participants as an important attractor, but with a caveat: it had to be within the 5 min travel time constraint. This makes it hard to implement on a large campus. Additionally, when visiting this central lunch location, people need to feel open to conversations with strangers. This means support within the organizational culture, creating an open and encouraging atmosphere, is important. At events, people come with the intention of meeting others and are therefore more open to start a conversation. This is likely why the addition of a conference center or other type of event space was often mentioned.

### 4.3. Practical Relevance

These results, in combination with the results presented in our previous paper [31] could be helpful to campus managers and designers as they provide a design concept and practical framework. This enables practitioners to identify possible interventions for an existing campus and prioritize those solutions. The results show that the 5-minute campus concept should be applied consistently and also be accompanied by a close study of the other key success factors. To further improve this framework, it would be recommended in future research to include other spatial analysis methods, e.g., space syntax to measure accessibility and other factors. 

### 4.4. Strenghts and Limitations

By building on our previous large-scale survey study, which included multiple Dutch university campuses, this study provides a more in-depth understanding of the motivators and constraints regarding unplanned meetings of campus users from both knowledge institutions as well as companies. As the statistical analysis of our previous paper shows, these results can be applied in general (there were no statistical differences between different campuses [31]). By looking at both services and location characteristics the cohesion of campus design is preserved, which helps to understand the complex relationship between these two from a user’s perspective. The time limits suggested in this paper have been confirmed by the focus group participant through a confirmatory question after analysis of the focus group results. The 5-minute concept creates a practical framework for campus design practitioners, clearly indicating which motivators and constraints need to be balanced to facilitate unplanned meetings on campus. Although this paper provides more insight and a practical framework, future studies could include larger participant groups to ensure the suggested time limits are appropriate for all campus users. As this study focused on experiences before the global pandemic, an evaluation of experiences after returning to campus and possible influence of using an online focus group method is recommendable. 

## 5. Conclusions

To further understand the responses in the previous survey performed by the authors [31], a sample of survey participants was questioned in five focus groups of 50% academic staff and 50% company employees on why some campus locations have more success in hosting unplanned meetings then others. The results were thematically analyzed. Participants indicated that apart from all other important themes, time was the main constraint. During a normal workday people simply do not leave their workplace unless they have a good reason, an attractor, to entice them to walk across the campus. It is, therefore, proposed that campus designers and directors should create a 5-minute campus: a campus where workplace interactions can be reached in under 5 min and everything related to taking a lunch or coffee break in around 5 min. Depending on how well they fit within a respondent’s work interest, events can be located further away, exceeding the 5-minute limit, although the length of the event and the anticipated knowledge gain, are also important factors. 

However, even on a 5-minute campus, other themes are important too: function (food, drinks, events, work, and facilities), space (distance, experience, accessibility, and characteristics), and organization (coherence, culture, and organization). These themes all play a role in the process of campus users negotiating constraints when there are sufficient attractors or motivators.

The findings suggest that when designed wisely—e.g., in alignment with user needs—a campus may actually stimulate spontaneous meetings between new people. Spaces and facility services can be used to do so, and—when deemed necessary—be refined or redesigned to enrich the campus as a social system. By doing so, this can potentially stimulate the social user interactions, which may be a starting point for new collaborations among peers as well as between university staff and on-campus companies. It all starts with a good coffee.

## Figures and Tables

**Table 1:** Focus group composition.

Focus Group	Knowledge Institution	Company
1	3	0
2	5	1
3	2	3
4	1	3
5	0	4
Total	11	11

**Table 2:** An overview of findings.

Survey Results	Focus Group Propositions	Additional Findings
Buildings with a low score did not seem to host fewer meetings. Often these meetings came from within the same building.	‘Even if the quality of the building where I work is low, I still prefer to stay here for interactions.’	Strong emphasis on time constraints. E.g., a long travel time within their own building is a barrier to visiting other locations.
Movement between buildings with a similar theme (e.g., energy).	‘The only reason to move to a different building is if there are people there with specific knowledge I need (e.g., companies or special expertise).’	People will not visit a building just because it has a (related) theme. An event or planned meeting is necessary for people to feel invited to visit. However, with a clear and attractive function (themed or not) will attract people to a certain location.
Coffee and lunch were important services	‘When I go somewhere for coffee or lunch, it is one of the main moments in which I have unexpected meetings.’	This is mostly true for unexpected meetings with people participants are already familiar with, but not likely for completely new contacts. There is a cultural barrier to start a conversation with a complete stranger (about work) when it is not framed by an event, planned meeting, or introduction by an acquainted co-worker.
Events and meetings were indicated as important services	‘The main reason I meet people outside my building is for an event or meeting.’	This was a strong attractor and culturally appropriate setting to start new conversations. There is a need for campus management to be involved in organizing these events, to create a lively campus. Spaces and facilities to support this also have to be made available.
		Outdoor spaces were largely missing from the survey results, but were emphasized in the focus groups as important spaces for unplanned meetings.

**Table 3:** Natural moments for spatial-temporal interactions.

Natural Moment	Travel Time	Function
Short break	<5 min	Coffee, toilet
Lunch break	Approx. min	Food court, restaurant
Meeting	>5 min ^1^	Event, planned meeting

^1^ Depending on length and content.

## Data Availability

Not applicable.
